# Differential impact of mitigation policies and socioeconomic status on COVID-19 prevalence and social distancing in the United States

**DOI:** 10.1186/s12889-021-11149-1

**Published:** 2021-06-14

**Authors:** Hsien-Yen Chang, Wenze Tang, Elham Hatef, Christopher Kitchen, Jonathan P. Weiner, Hadi Kharrazi

**Affiliations:** 1 Department of Health Policy & Management, Johns Hopkins Bloomberg School of Public Health, Baltimore, Maryland USA; 2 Center for Drug Safety and Effectiveness, Johns Hopkins University, Baltimore, Maryland USA; 3 Center for Population Health Information Technology, Johns Hopkins University, Baltimore, Maryland USA; 4 Department of Epidemiology, Harvard T.H. Chan School of Public Health, Boston, Massachusetts USA; 5 Division of Health Sciences Informatics, Johns Hopkins School of Medicine, Baltimore, Maryland USA

**Keywords:** COVID-19 policy, COVID-19 prevalence, Area deprivation index, Social determinants of health, Social distancing index, Comparative interrupted time-series analysis, Stay-at-home order

## Abstract

**Background:**

The spread of COVID-19 has highlighted the long-standing health inequalities across the U.S. as neighborhoods with fewer resources were associated with higher rates of COVID-19 transmission. Although the stay-at-home order was one of the most effective methods to contain its spread, residents in lower-income neighborhoods faced barriers to practicing social distancing. We aimed to quantify the differential impact of stay-at-home policy on COVID-19 transmission and residents’ mobility across neighborhoods of different levels of socioeconomic disadvantage.

**Methods:**

This was a comparative interrupted time-series analysis at the county level. We included 2087 counties from 38 states which both implemented and lifted the state-wide stay-at-home order. Every county was assigned to one of four equally-sized groups based on its levels of disadvantage, represented by the Area Deprivation Index. Prevalence of COVID-19 was calculated by dividing the daily number of cumulative confirmed COVID-19 cases by the number of residents from the 2010 Census. We used the Social Distancing Index (SDI), derived from the COVID-19 Impact Analysis Platform, to measure the mobility. For the evaluation of implementation, the observation started from Mar 1st 2020 to 1 day before lifting; and, for lifting, it ranged from 1 day after implementation to Jul 5th 2020. We calculated a comparative change of daily trends in COVID-19 prevalence and Social Distancing Index between counties with three highest disadvantage levels and those with the least level before and after the implementation and lifting of the stay-at-home order, separately.

**Results:**

On both stay-at-home implementation and lifting dates, COVID-19 prevalence was much higher among counties with the highest or lowest disadvantage level, while mobility decreased as the disadvantage level increased. Mobility of the most disadvantaged counties was least impacted by stay-at-home implementation and relaxation compared to counties with the most resources; however, disadvantaged counties experienced the largest relative increase in COVID-19 infection after both stay-at-home implementation and relaxation.

**Conclusions:**

Neighborhoods with varying levels of socioeconomic disadvantage reacted differently to the implementation and relaxation of COVID-19 mitigation policies. Policymakers should consider investing more resources in disadvantaged counties as the pandemic may not stop until most neighborhoods have it under control.

**Supplementary Information:**

The online version contains supplementary material available at 10.1186/s12889-021-11149-1.

## Background

In the US, the total number of confirmed COVID-19 cases has skyrocketed from 30 patients on Mar 1st, 2020 to over 6.4mil on Sep 11th with total deaths exceeding 192 k [1]. With no proven high-efficacy drug treatments, physicians can only provide supportive care for COVID-19 patients [2]. The lack of a definite treatment has also propelled healthcare professionals and policymakers to further focus their efforts on preventing the transmission of the disease.

Various approaches to slowing down the COVID-19 transmission have been recommended, with a focus on either self-protection or limited in-person contacts [3, 4]. In the absence of a universal mitigation policy by the US federal government, state and local governments have implemented a range of social distancing policies to restrict in-person contacts and limit mobility, such as restricting dine-in at restaurants, closing non-essential business, and banning large gatherings [3–5]. Among a wide range of mitigation policies, the stay-at-home (SAH) order has been the most restrictive policy with early studies documenting various levels of effectiveness of such policy [5–9].

Forty states, including the District of Columbia, implemented the state-wide SAH order in Mar and Apr 2020 during an initial surge of COVID-19 transmission [10]. Despite the effectiveness of SAH order in reducing the COVID-19 transmission [4–7, 9], its impact on economic activity was deemed detrimental [4]. Consequently, as COVID-19 transmission started to slow down between Apr and Jun 2020, 38 out of 40 states lifted their SAH order [10]. Evaluating the impact of the SAH orders, both implementation and relaxation of the orders, on COVID-19 transmission would provide useful information given the recent resurgence of COVID-19 transmissions across the US [1].

The spread of COVID-19 has highlighted the established health inequalities across the US. For example, neighborhoods with higher income inequality, a higher proportion of racial or ethnic minorities, lower median family income, and higher unemployment rates were associated with higher rates of COVID-19 transmission, hospitalization, and death [11–14]. Similar associations were observed at the individual level [15]. Studies also found that residents in lower-income neighborhoods faced barriers to practicing physical distancing or following mobility restriction, particularly given the need to work outside the home [16–22]. These findings raise the question about the differential impact of the implementation and lifting of SAH orders on neighborhoods with varying levels of disadvantage and uneven levels of COVID-19 burden.

This study assesses the effectiveness of SAH policy on decreasing the COVID-19 transmission across neighborhoods with different levels of socioeconomic disadvantage represented by the Area Deprivation Index (ADI). We describe the differential impact of the implementation and lifting of SAH order on COVID-19 prevalence and residents’ mobility across counties with different levels of ADI (the highest/Q4, high/Q3, low/Q2, the lowest/Q1) [23–25]. We perform additional analyses stratified by the population density to evaluate the potential interaction between SAH policy and population density in affecting both mobility and COVID-19 incidence [23–25].

## Methods

### Data

We compiled data from several data sources for this analysis. We derived the daily number of cumulative confirmed COVID-19 cases at the county level from the COVID-19 dashboard by the Center for Systems Science and Engineering (CSSE) at Johns Hopkins University [4]. We measured the mobility at the county level using the Social Distancing Index (SDI). We derived the daily SDI from the COVID-19 Impact Analysis Platform (CIAP) created by the Maryland Transportation Institute at the University of Maryland [26]. We determined the socioeconomic status of a county using ADI [27, 28]. We derived the county-specific characteristics, such as the percentage of the elderly, from the US Census American Community Survey (ACS) data of 2018. We derived the population size of each county from the 2010 Census. We did not obtain institutional review board approval due to the use of publicly available, de-identified data, per usual institutional policy.

### Social distancing index (SDI)

SDI was used to represent mobility at the county level in our study. SDI uses the location data from anonymized mobile devices that are integrated with geographical population data. SDI for each state and county was derived from information such as percentage of people who are staying home, the average number of trips per person and average distance traveled by each person [26]. SDI, ranging from 0 to 100, represented the extent residents/visitors would practice social distancing: “0” indicated no social distancing in the community while “100” indicated all residents were staying at home and no visitors were entering the county. The higher the SDI, the lower the mobility. The mean national county-level SDI was 34.1 on March 7th when SDI data first became available.

### Area deprivation index (ADI)

ADI is a widely used measure of socioeconomic disadvantage at various geographical levels [27–29]. We took the following steps to calculate the ADI using the latest Census data: (1) we used 71 independent census variables from 5-year estimate American Community Survey data of 2018 to construct 17 ADI grouped variables, and these census variables include: education (such as the percentage of population aged ≥25 y with at least a high school diploma), income (such as median family income), housing condition (such as percentage of owner-occupied housing units) and employment (such as percentage of employed persons aged ≥16 y in white-collar occupations); (2) we summed up weighted 17 ADI components as total scores using Singh et al. methodology [29]; and, (3) we normalized total scores to have a mean of 100 and a standard deviation of 20. We constructed the ADI raw scores for 52 states (i.e., continental states, Alaska, Hawaii, and Puerto Rico) at the county level and assigned each county to one of four equally-sized ADI levels based on its ADI score (the highest/Q4: 76th–100th percentile of the ADI score, high/Q3: 51st to 75th percentile, low/Q2: 26th to 50th percentile, and the lowest/Q1: 1st to 25th percentile). The higher the ADI, the higher level of disadvantage in a county.

### State stay-at-home order

Out of 51 states including the District of Columbia, 11 states did not implement SAH order (Arkansas, Connecticut, Iowa, Kentucky, Nebraska, North Carolina, Oklahoma, South Dakota, Texas, Utah, and Wyoming) and 13 states did not lift SAH order as of Aug 3rd 2020 (the aforementioned 11 states plus California and New Mexico) [10]. To keep the inference population consistent between analyses of SAH implementation and lifting effects, we restricted all of our analyses to the 38 states with both implementing and lifting SAH orders, covering 2087 counties and approximately 212 out of 338 (62.5%) million Americans. 

### Outcomes

We included two outcomes in the analysis: COVID-19 prevalence and SDI. For each county, we calculated the daily COVID-19 prevalence by dividing the daily number of confirmed COVID-19 cases by the number of residents. We derived the daily SDI at the county level directly from the CIAP. To minimize the fluctuations in the derived SDI, we used 7-day simple-averaged SDI.

### Time segments

We used two different observation periods for the comparative analysis: (1) To assess the effect of the SAH implementation, we included the observation period starting from March 1st to 1 day before the SAH lifting date; and, (2) To assess the effect of the SAH lifting, we included the observation period starting from 1 day after the SAH implementation date to Jul 5th (data cutoff date) of each county. We obtained the dates of implementation and lifting of SAH order at the state level from the “COVID-19 US state policy database” project by Raifman et al. at the Boston University School of Public Health (reviewed on Aug 3rd, 2020) [10]. We assigned the counties within the same state to the same dates of SAH implementation and lifting.

### Analyses

**Fig. 1:**
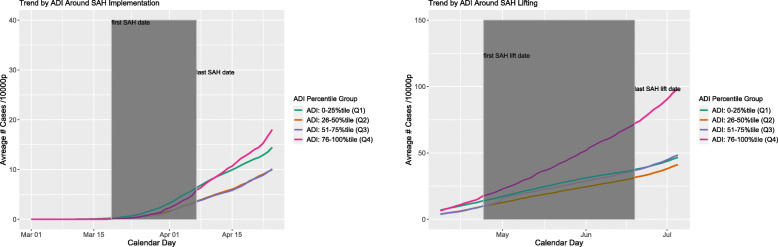
Average prevalence by ADI groups over calendar time

**Fig. 2:**
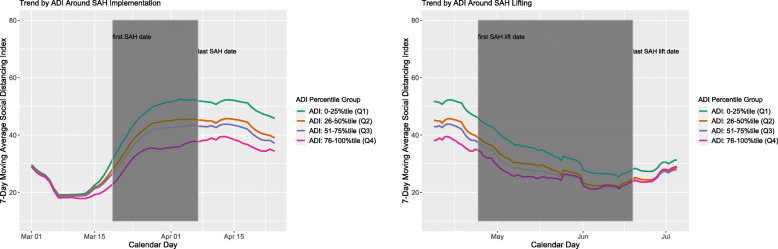
Seven-day moving average SDI by ADI Groups over calendar time

We first described and compared the county characteristics within each of the four ADI levels using chi-squared tests for categorical variables and Kruskal-Wallis test for continuous variables. To visualize the change in trends of county-level COVID-19 prevalence (Fig. 1) and SDI (Fig. 2), we plotted these two outcomes in separate figures by calendar time, averaging over counties of the same ADI level. Within each outcome/figure, we produced separate plots for period around implementation and lifting of SAH order due to potential scale differences in outcomes. As each county might have different dates of SAH implementation and lifting, we also added time frame indicating the earliest and last policy date to each plot; for example, the shaded areas in Fig. 1 refer to the range of SAH policy implementation dates across counties.

To compare the policy impact across counties at four ADI levels, we adopted a comparative interrupted time series (ITS) framework. The comparative ITS analysis has been used to evaluate the policy impact on outcomes between the case (with the policy implementation) and the control (without the policy implementation) [30, 31]. In our study, these observations provided a comparative change of daily trends in the outcomes between counties at three higher ADI levels (Q2-Q4) and those at the lowest ADI level (Q1; the reference group) before and after the implementation/lifting of SAH order. In order to take into account either the anticipated or delayed effect of SAH order, we chose all actual interrupted time points after examining plots of empirical trends of COVID-19 prevalence and SDI at SAH implementation and lifting index date (Additional file 1: Appendix Figure 1a and b) [32]. Thus, we used the following dates to evaluate the respective policy’ impact on COVID-19 prevalence: 5 days after SAH implementation and 20 days after SAH lifting date. Similarly, we used 15 days before SAH implementation and 40 days before SAH lifting to evaluate their respective impact on change in SDI. One advantage of such design is that it is not prone to time-independent confounding (such as gender and race) or other confounding (such as age and income) that is unlikely to change over a short period of time [12].

We used a linear mixed-effect model to quantify the effect of implementing and lifting SAH order on each outcome. Our parameter of interest was the interaction of the ADI level indicator and the post-policy day indicator, representing the effect of the policies on the rate of change (trend) from the pre-policy to post-policy periods across four ADI levels. For each county, we included random effect terms for intercept (e.g., baseline COVID-19 prevalence), pre-policy outcome trend (e.g., COVID-19 prevalence trend before SAH implementation) and post-policy outcome trend (e.g., difference in COVID-19 prevalence trend after SAH implementation). We also included day of week to adjust for its time-varying effect. All statistical tests were two-sided with alpha level of 0.05.

All analyses were conducted using R 4.0.

## Results

### Characteristics of counties by ADI level

We included 2087 counties in the analysis, with 521 or 522 counties in each level of ADI. The observation time for SAH implementation ranged from 86.2 days among counties with ADI Q1 level to 95.8 days among counties with ADI Q3 level. The observation time for SAH lifting was about the same, ranging from 89.5 days among counties with ADI Q1 level to 93.2 days among counties with ADI Q4 level. We observed statistically significant differences in characteristics of counties across four levels of ADI (Table 1). As the county’s ADI level decreased, the population size, the population density, the median family income and the percentage of residents with at least high school diploma increased while the percentages of families in poverty and residents unemployed decreased. For example, the mean number of residents increased from 37,381 in ADI Q4 level to 176,972 in ADI Q1 level. The mean age ranged from 40.7 years old among counties with ADI Q4 level to 42.3 years old among counties with ADI Q2 level.

**Table 1:** Characteristics of counties by the Area Deprivation Index level

ADI Level	Lowest / Q1	Low / Q2	High / Q3	Highest / Q4	All Levels
# of counties	522	522	522	521	2087
Observation time / SAH Implementation, days	95.7 (14.2)	95.8 (13.0)	94.0 (13.4)	86.2 (10.5)	92.9 (13.4)
Observation time / SAH Lifting, days	93.2 (3.8)	92.9 (4.6)	92.3 (5.0)	89.5 (4.6)	92.0 (4.8)
# of residents	176,972 (276,383)	120,052 (356,081)	70,334 (169,653)	37,381 (110,126)	101,215 (252,467)
Density, count per squared mile	43,367 (98,615)	25,825 (96,300)	15,697 (42,369)	13,972 (68,397)	24,726 (80,604)
Density - Low: 1st – 10th	65 (12.5%)	73 (14.0%)	47 (9.0%)	24 (4.6%)	209 (10.0%)
Density - Medium: 11th – 90th	349 (66.9%)	394 (75.5%)	450 (86.2%)	476 (91.5%)	1669 (80.0%)
Density - High: 91st – 100th	108 (20.7%)	55 (10.5%)	25 (4.8%)	20 (3.8%)	208 (10.0%)
Median age, years	41.5 (5.3)	42.3 (5.6)	42.1 (5.5)	40.7 (4.6)	41.7 (5.3)
% elderly	17.9% (5.3%)	19.2% (4.6%)	19.0% (4.3%)	17.6% (3.4%)	18.4% (4.5%)
Median family income, $	83,408 (15,914)	65,721 (5581)	57,718 (4691)	47,992 (5932)	63,717 (15,940)
% families in poverty	6.1% (1.9%)	9.0% (2.0%)	11.9% (2.3%)	18.2% (4.9%)	11.3% (5.4%)
% people unemployed	4.3% (1.4%)	5.1% (1.2%)	6.3% (1.9%)	8.5% (3.3%)	6.0% (2.7%)
% people with at least high school education	92.3% (2.7%)	89.3% (2.9%)	86.3% (3.7%)	79.9% (4.5%)	87.0% (5.8%)
COVID-19 prevalence (# cases per 10,000 people)					
On March 1st	0.0 (0.2)	0.0 (0.1)	0.0 (0.0)	0.0 (0.0)	0.0 (0.1)
At SAH implementation	1.7 (3.3)	1.0 (1.8)	0.9 (1.9)	2.6 (5.5)	1.6 (3.5)
At SAH lifting	26.6 (37.8)	19.4 (35.9)	21.9 (59.6)	28.5 (42.6)	24.1 (45.1)
Social Distancing Index					
On March 1st	35.3 (7.5)	34.2 (7.1)	33.6 (7.1)	33.2 (6.9)	34.1 (7.2)
At SAH implementation	50.6 (11.7)	44.0 (11.2)	40.6 (10.8)	32.1 (11.3)	41.8 (13.1)
At SAH lifting	30.4 (11.6)	24.7 (10.0)	23.3 (9.2)	21.0 (8.7)	24.8 (10.5)

Numbers represent mean while those within the brackets represent standard deviation

*ADI* Area Deprivation Index, *SAH* Stay at Home

A U-shape relationship was observed between ADI level and the COVID-19 prevalence (Table 1). Counties with ADI Q4 or Q1 level had much higher COVID-19 prevalence on both SAH implementation and lifting dates than those with Q3 or Q2 level. A dose-response relationship was identified between the ADI level and SDI: as the ADI level increased (i.e., more disadvantaged neighborhoods), SDI decreased (more mobility) on both SAH order implementation and lifting dates.

### Average county-level COVID-19 prevalence by the ADI level over calendar time

Figure 1 depicts the average county-level COVID-19 prevalence by the ADI level over calendar time. At the SAH implementation, counties with ADI Q4 level had the largest increase in the slope of COVID-19 prevalence from the pre- to post-SAH implementation, while counties with ADI Q1 level seemed to have the smallest increase. Eventually, counties with ADI Q4 level had the highest absolute COVID-19 prevalence rate. Counties with ADI Q3 or Q2 level had similar prevalence rates across the entire observation period. Similarly, at the SAH lifting, counties with ADI Q4 level also had the largest increase in the slope of COVID-19 prevalence from the pre- to post-SAH lifting. Counties with other three ADI levels had similar trends before and after SHA lifting. Same plot further stratified by population density, a potential effect modifier, is presented in Additional file 1: Appendix Figure 2a, where some idiosyncrasy is observed for the trends among counties with top 10 percentile population density.

### Average county-level SDI by the ADI level over calendar time

Figure 2 shows the average county-level SDI by the ADI level over calendar time. SDI showed more fluctuations than COVID-19 prevalence. SDI across all counties increased before, stayed flat during, and started to decrease after the SAH implementation. SDI across all counties decreased before, continued to decrease during, and started to increase after the SAH lifting. In counties with higher ADI levels, SDI was lower over the entire observation period. Same plot further stratified by population density, a potential effect modifier, is presented in Additional file 1: Appendix Figure 2b.

### Effect of SAH implementation

**Fig. 3:**
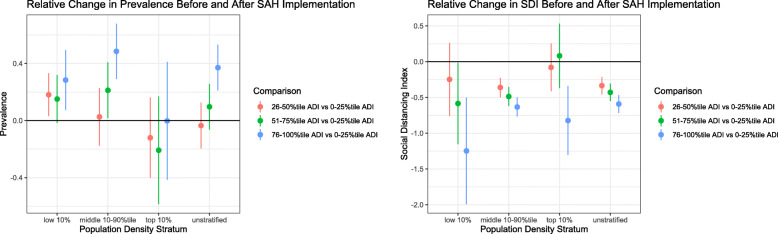
Differential effect of stay-at-home policy implementation on prevalence and SDI

Following the implementation of the SAH order, the counties with ADI Q4 level experienced a statistically significantly relative increase in the daily trend of COVID-19 prevalence (0.371 prevalence/day, 95% Confidence Interval (CI) 0.211 to 0.532) compared to those with ADI Q1 level. The counties with ADI Q2 or Q3 level did not experience such significantly relative differences (Fig. 3 & Additional file 1: Appendix Table 1).

Counties with the non-Q1 ADI levels experienced statistically significantly relative reductions in the daily trend of SDI compared to those with ADI Q1 level. For example, a daily relative decline of 0.592 SDI/day (95% CI − 0.717 to − 0.467) were detected when comparing the counties with ADI Q4 to ADI Q1 level. Compared to the counties with ADI Q1 level, such relative reduction was 0.335 SDI/day (95% CI − 0.454 to − 0.215) for those with ADI Q2 level and 0.429 SDI/day (95% CI − 0.549 to − 0.308) for those with ADI Q3 level (Fig. 3 & Additional file 1: Appendix Table 1).

Results from the stratified analyses by the population density were mostly similar to those from the unstratified analysis of the entire study population with some exceptions. For example, in the analyses of the daily prevalence, no statistically significantly associations were observed among high-density counties while the significant association was also observed comparing ADI Q2 to ADI Q1 level (0.181 prevalence/day, 95% CI 0.031 to 0.331) among low-density counties and ADI Q3 to ADI Q1 level (0.212 prevalence/day, 95% CI 0.016 to 0.408) among medium-density counties. In the analyses of daily SDI, no significant associations were observed comparing ADI Q2 to ADI Q1 level among low-density counties and comparing ADI Q2 or Q3 to ADI Q1 level among high-density counties (Fig. 3 & Additional file 1: Appendix Table 1).

### Effect of SAH lifting

**Fig. 4:**
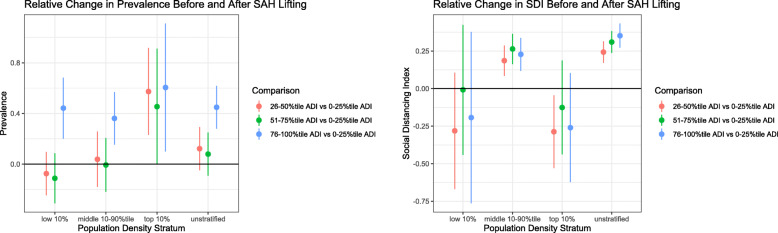
Differential effect of stay-athome policy lifting on prevalence and SDI

Following the lifting of the SAH order, the counties with ADI Q4 level experienced a statistically significantly relative increase in the daily trend of COVID-19 prevalence (0.449 prevalence/day, 95% CI 0.280 to 0.618) compared to those with ADI Q1 level. Counties with ADI Q2 or Q3 level did not experience such significantly relative differences (Fig. 4 & Additional file 1: Appendix Table 2).

Counties with ADI non-Q1 levels (i.e., Q2, Q3 and Q4) experienced statistically significantly relative increases in the daily trend of SDI compared to those with ADI Q1 level. For example, there was a daily relative increase of 0.352 SDI/day (95% CI 0.272 to 0.433) comparing the counties with ADI Q4 to Q1 level. Compared to the counties with ADI Q1 level, such relative increase was 0.243 SDI/day (95% CI 0.171 to 0.315) for those with ADI Q2 level and 0.310 SDI/day (95% CI 0.237 to 0.383) for those with ADI Q3 level (Fig. 4 & Additional file 1: Appendix Table 2).

Results from the density-stratified analyses were similar to those from the unstratified analysis of the entire study population for the outcome of COVID-19 prevalence but not SDI. For example, no statistically significant relative difference was detected in daily SDI comparing low-density counties with the three ADI non-Q1 levels to those with Q1 level. The only statistically significant relative difference among high-density counties was the daily relative reduction of 0.287 SDI/day (95% CI − 0.529 to − 0.045) comparing ADI Q2 to Q1 level (Fig. 4 & Additional file 1: Appendix Table 2).

## Discussion

COVID-19 has affected US neighborhoods and communities disparately, with minorities and less resourceful communities taking most of the burden. Despite these initial findings, evidence on the effect of neighborhood-level disparities on the effectiveness of SAH policy and COVID-19 transmission is still lacking. Our study revealed the role of social disparities in compliance with the SAH order implementation and relaxation and the impact on COVID-19 transmission across neighborhoods. When compared to the counties with ADI Q1 level, we found a comparative increase in the COVID-19 rate among counties with ADI Q4 level and relatively less increase in the social distancing among counties with higher ADI after the SAH order was implemented. After the lifting of the SAH order, compared to the counties with ADI Q1 level, we found a comparative increase in the COVID-19 rate among counties with ADI Q4 level and relatively more social distancing among counties with higher ADI. In short, mobility of counties with ADI Q4 level (i.e., most disadvantaged counties) was least impacted by SAH implementation and lifting compared with Q1 level, but experienced the worst relative increase in COVID-19 infection after both SAH implementation and lifting.

Our study found that the COVID-19 prevalence was higher not only in the most disadvantaged but also the least disadvantaged counties. While the higher COVID-19 prevalence among the poorest regions is not new [33–35], we did not expect the COVID-19 prevalence would be also higher among the richest counties; such results are consistent even when stratified by population density level (results not shown). The higher prevalence of COVID-19 among the least disadvantaged counties can be the result of the more COVID-19 testing available to them at the early stage of the pandemic; the more the testing, the more the cases identified. Various studies have shown the wide disparity in COVID-19 testing and found that the COVID-19 testing rates were lower among Black and poor residents [36–40].

The differential responses to the implementation of the SAH order by neighborhoods’ levels of socioeconomic disadvantage have been reported in previous studies, but most studies focused on the mobility rate among residence of different neighborhoods [16, 17, 20, 21]. One study found that physical distancing orders were associated with less increase in staying home in low-income vs. high-income neighborhoods (1.5% vs 2.4%) [17]. Another study found that areas with fewer resources had more subway use in New York City [20]. These findings were consistent with ours. It is likely that residents in more disadvantaged neighborhoods (represented by higher ADI levels) had fewer resources to stay at home. For example, they may still need to work outside the home such as essential workers. Consequently, such comparative increase in residents’ mobility may lead to an increase in the in-person contacts, which eventually may have resulted in the relative increase in the COVID-19 prevalence in disadvantaged neighborhoods (i.e., high ADI), as we observed in this study. Moreover, social distancing might be more challenging in socioeconomically disadvantaged neighborhoods with high housing density and overcrowding [41–43]. Additionally, communities that are mainly comprised of economically challenged households are more likely to be exposed to COVID-19 due to their overrepresentation in the low-wage, essential work at the front lines [41]. Notably, our result differs from a recent Italian study where it found that mobility contraction is stronger in municipalities where inequality is higher and income per capita is lower [44]. We suspect such difference in finding has largely to do with the availability and readiness of welfare systems.

We have not identified any research on the impact of lifting the SAH orders. The SAH order lifting might have increased social and entertainment activities, with residents in the low ADI neighborhoods (i.e., less disadvantaged) having the means to socialize compared to those in high ADI neighborhoods. On the other hand, more people in the high ADI neighborhoods (i.e., higher disadvantaged) may have lost their jobs during the pandemic (decrease in mobility given no need to go out to work) or have to continue working regardless of the SAH order, such as essential workers (no change in mobility). Thus, we observed relatively increases in social distancing after such lifting among higher disadvantaged neighborhoods. However, even with the relatively decreasing trend in mobility, neighborhoods with the highest ADI still experienced a relatively increasing rate of COVID-19 cases. It is possible that residents in less disadvantaged neighborhoods practiced self-protection measures better (e.g., able to purchase face masks or hand sanitizers), as various studies have shown that higher-resource neighborhoods were associated with less COVID-19 transmission [11–14]. Our findings also align with another study that pointed out health disparities may play a more important role in COVID-19 transmission than government interventions (such as SAH order) and community-level compliance to such interventions [13].

Our study had some limitations. First, this was an ecological study and results from the aggregated data might not be generalizable to individuals. Second, some counties implemented various policies aiming at reducing COVID-19 transmission during our study period (e.g., shelter-in-place or stay-at-home orders; restricting dine-in at restaurants; closing nonessential business such as bars, entertainment venues, and gyms; banning large social gatherings; and closing public schools) and the strength of implementing these policies may also vary across counties. Although we adopted the random intercept method to control for the fixed state effects, our results may not be attributed to the SAH order alone. Third, we used ADI to represent the disadvantage level of a neighborhood (representing the overall social determinants of health), and SDI to represent mobility. While both measures, especially ADI, have been examined extensively, these measures may still not capture the concepts they aim to represent completely. For example, SDI is based on mobile device data; it may not capture the mobility in the extremely disadvantaged counties with low use of smartphones and/or low penetration of internet. Fifth, due to the limitation in the flexibility allowed for linear mixed effect model, we only modeled county-level random intercept and random slopes for pre-SAH/post-SAH implementation and did not explicitly model within-state correlation using a state-level random intercept. Sixth, initial inadequate testing during the pandemic might disproportionally affect counties with higher ADI level, biasing down the true prevalence level at the beginning of the pandemic. Given the lack of valid testing data, we cannot evaluate the impact of inadequate testing on our finding. Lastly, we may not be able to generalize the results to all counties in the US as 1055 counties from 13 states were not included in this study given lack of one or both SAH policy. For example, even though similarity existed between counties included in and excluded from our study (counties included vs counties excluded: % elderly 18.4% vs 18.3%; % families in poverty 11.3% vs 11.1%), some differences could be observed (% people employed 6.0% vs 5.2%; # residents 101,215 vs 117,262).

## Conclusions

Our study, despite having limitations, showed that neighborhoods with varying levels of disadvantage reacted differently to the implementation and relaxation of COVID-19 mitigation policies. Those with the highest ADI (i.e., most disadvantaged counties) observed the largest relative increases after both the SAH implementation and lifting. Policymakers should consider investing more resources in these disadvantaged counties to help them contain the COVID-19 transmission in future SAH implementations and liftings, as the pandemic may not stop until most, if not all, neighborhoods have it under control.

## Supplementary Information

**Additional file 1.**

## Data Availability

All data generated or analyzed during this study are included as a supplementary compressed csv file named “df_SAH_policy_on_covid19.zip”. *COVID-19 cases / Open to public access.* Dong E, Du H, Gardner, L. COVID-19 Data Repository by the Center for Systems Science and Engineering (CSSE) at Johns Hopkins University. Baltimore, MD: GitHub; Date last modified June 9, 2020. Available at: https://github.com/CSSEGISandData/COVID-19. Accessed June 9, 2020. *Social Distancing Index / Open to public access.* Zhang L, Ghader S, Pack ML, Xiong C, Darzi A, Yang M, Sun Q, Kabiri A, Hu S: AN INTERACTIVE COVID-19 MOBILITY IMPACT AND SOCIAL DISTANCING ANALYSIS PLATFORM. medRxiv 2020:2020.2004.2029.20085472. Available at: https://data.covid.umd.edu. Accessed June 9, 2020. *COVID-19 US state policy database / Open to public access.* Raifman J, Nocka K, Jones D, Bor J, Lipson S, Jay J, and Chan P. (2020). “COVID-19 US state policy database.” Available at: https://www.tinyurl.com/statepolicies. Accessed August 7, 2020.

## Abbreviations

ACS: American Community Survey
ADI: Area Deprivation Index
ITS: Interrupted time series
CIAP: COVID-19 Impact Analysis Platform
CSSE: Center for Systems Science and Engineering
SAH: Stay-at-home
SDI: Social Distancing Index
